# Clinical testing on SARS-CoV-2 swab samples using reverse-transcription loop-mediated isothermal amplification (RT-LAMP)

**DOI:** 10.1186/s12879-022-07684-w

**Published:** 2022-08-18

**Authors:** Meng Yee Lai, Fatma Diyana Mohd Bukhari, Nur Zulaikha Zulkefli, Ilyiana Ismail, Nur Izati Mustapa, Tuan Suhaila Tuan Soh, Afifah Haji Hassan, Kalaiarasu M. Peariasamy, Yee Leng Lee, Jeyanthi Suppiah, Ravindran Thayan, Mohd Khairi Mat Isa, Nur Zafirah Abdul Wahid, Yee Ling Lau

**Affiliations:** 1 Department of Parasitology, Faculty of Medicine, Universiti Malaya, 50603 Kuala Lumpur, Malaysia; 2 Department of Pathology, Hospital Sungai Buloh, Ministry of Health, Kuala Lumpur, Malaysia; 3 Institute for Clinical Research, National Institutes of Health, Ministry of Health, Kuala Lumpur, Malaysia; 4 Clinical Research Centre, Hospital Sungai Buloh, Ministry of Health, Kuala Lumpur, Malaysia; 5 Virology Unit, Infectious Disease Research Centre, Institute for Medical Research, National Institutes of Health, Ministry of Health, Kuala Lumpur, Malaysia; 6 Selia-Tek Holdings Sdn Bhd, Lot 18, Jalan Teknologi 3/5, Taman Sains Selangor, 47810 Kota Damansara, Selangor Malaysia

**Keywords:** Diagnosis, RT-LAMP, SARS-CoV-2, Phenol red, pH sensitive indicator

## Abstract

**Background:**

High cost of commercial RNA extraction kits limits the testing efficiency of SARS-CoV-2. Here, we developed a simple nucleic acid extraction method for the detection of SARS-CoV-2 directly from nasopharyngeal swab samples.

**Methods:**

A pH sensitive dye was used as the end point detection method. The obvious colour changes between positive and negative reactions eliminates the need of other equipment.

**Results:**

Clinical testing using 260 samples showed 92.7% sensitivity (95% CI 87.3–96.3%) and 93.6% specificity (95% CI 87.3–97.4%) of RT-LAMP.

**Conclusions:**

The simple RNA extraction method minimizes the need for any extensive laboratory set-up. We suggest combining this simple nucleic acid extraction method and RT-LAMP technology as the point-of care diagnostic tool.

**Supplementary Information:**

The online version contains supplementary material available at 10.1186/s12879-022-07684-w.

## Introduction

Coronavirus disease 2019 (COVID-19), caused by the most recently discovered coronavirus, severe acute respiratory syndrome coronavirus 2 (SARS-CoV-2) has hit the whole world since December 2019. Mass testing and identification of infected individuals are of utmost importance in the ongoing COVID-19 pandemic. Easy and rapid laboratory diagnosis are needed to control this pandemic. This study aims to simplify the current method used to diagnose COVID-19 as well as suggest better sample collection and RNA extraction methods. At present, real-time reverse transcription polymerase chain reaction (RT-qPCR) method remains the gold standard and most reliable detection method to detect the virus. However, PCR-based detection method is laborious, expensive, and time consuming as it requires special instruments, supply-limited reagents, and well-trained personnel [1, 2]. An alternative to RT-qPCR is reverse transcription loop-mediated isothermal amplification (RT-LAMP), an assay that can detect nucleic acid in a short time using 4 to 6 specially designed primers that hybridize with 6 to 8 regions of the target gene, resulting in high specificity [3]. RT-LAMP colorimetric assay enables rapid and easy interpretation of results that requires only an isothermal heat source [4]. This makes it simpler, cheaper, and time-efficient compared to other molecular methods [3, 5].

Here, we used a modified Chelex 100 Resin concentration method coupled with RT-LAMP colorimetric test on nasopharyngeal swab samples in viral transport media (VTM) by amplifying the *N* gene which codes the nucleocapsid region of SARS-CoV-2. The RNA was extracted using chelating resin without a further purification step. The extracted RNA served as a template for the RT-LAMP assay. Phenol red, a pH sensitive colorimetric dye was used as the colour indicator [6]. Positive amplification of the target sequence resulted in a colour change from pink to yellow.

## Methods

### Sample sources

Hospital Sungai Buloh (HSB) and Institute for Medical Research (IMR), Malaysia, provided a total of 260 fresh nasopharyngeal swab samples, in 250 µL of VTM (Additional file 1: Table S1). Prior to that, the swab samples were heat-inactivated at 65 °C for 1 h. Of 260 samples, 150 samples were confirmed positive for SARS-CoV-2 (HSB, n = 60 and IMR, n = 90) by RT-qPCR with Ct value range from 12.71 to 38.80, while 110 samples were reported negative (HSB, n = 50 and IMR, n = 60). The RNA extraction kit and RT-qPCR kit involved in this study were QIAamp Viral RNA Mini Kit (Hilden, Germany) and SuperScript™ III Platinum™ One-Step qRT-PCR Kit (Thermo Fisher Scientific, Massachusetts, United States), respectively. This study was approved by UMMC Medical Ethics Committee (202041-8418) and Malaysian Ministry of Health Medical Research Ethics Committee (MREC) (NMRR-20-2344-56994).

### RNA preparation

RNA extraction was carried out by using Chelex 100 Resin extraction protocol adopted from Janíková et al. and Perez et al. with minor modifications [7, 8]. Chelex 100 Resin (Biorad Laboratories, USA) was weighed and diluted with 1× TE Buffer (pH 8) (Promega Corp., USA) in 30% concentration. The 30% (w/v) Chelex-TE was vortex vigorously for 10 s and kept in 4 °C for proper storage. In sterile conditions, 30 µL of samples was mixed with 45 µL of 30% Chelex-TE by pipetting up and down thrice. The reaction was incubated in 98 °C for 2 min, followed by another 2 min incubation on ice. After that, the sample was spun down for 1 min. The supernatant was transferred into a new tube and the pellet was discarded. Sodium acetate (NaAc) with pH 5.3 was added into the supernatant with final concentration 0.3 M. Then, 3 volume of ice-cold ethanol (225 µL) was included into the reaction and vortexed for 5 s. The tube was spun down for 5 min to collect the RNA pellet at the bottom. The supernatant was discarded and the pellet was subjected to air-dry for 5–10 min. The pellet was re-suspended using 10 µL of 1× TE Buffer, followed by vortex for 30 s. The suspension was served as a template in RT-LAMP assay.

### RT-LAMP assay

**Fig. 1:**
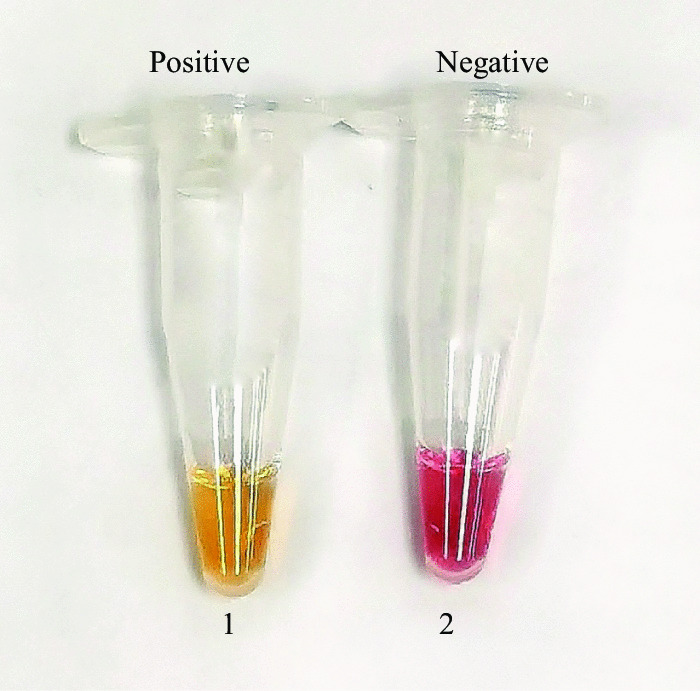
Visualization of the RT-LAMP with the colour change of phenol red from pink (negative) to yellow (positive). Tube 1: Positive reaction; Tube 2: Negative reaction

The extracted RNAs were amplified using nucleocapsid (N) gene, targeting on N1 region. Primer-Explorer V4 software (Eiken Chemical Co., Ltd., Tokyo, Japan) was used to design the primers (Table 1) and it has been reported previously [9]. The RT-LAMP assay was conducted in a total of 12.5 μL reaction mixture, which be made up of 3.8 μL RNAse free water, 1.25 µL 10× low strength buffer (pH 8.3), 0.75 µL magnesium sulphate MgSO_4_ (100 mM), 0.175 µL of each dNTPs (dATP, dCTP, dGTP and dTTP 100 mM each), 1.9 µL primer mix (consisting of 40 pmol FIP and BIP each, 10 pmol of FLP and BLP each, 5 pmol of F3 and B3 each), 0.75 µL *Bacillus stearothermophilus* (*Bst*) 2.0 WarmStart DNA polymerase, 0.15 µL WarmStart RTx reverse transcriptase, 0.5 µL RNase inhibitor (0.5 U/μL) (NEB, Ipswich, United States), 0.5 µL guanidinium hydrochloride (GuHCl) (1 M), 0.2 µL phenol red (10 mM) and 2 µL RNA template. The reaction was incubated in 50 °C for 10 min, followed by 65 °C for 1 h and lastly, inactivated at 80 °C for 2 min. This RT-LAMP assay was performed using heating block (Hangzhou Ruicheng Instrument Co., Ltd., Hangzhou, China). Phenol red was used for direct visual detection of the end product. The incubation process was monitored closely for every 10 min (up until 1 h) in order to identify the color changes. Yellow color indicates a positive sample, whereas negative reactions will remain as pink (Fig. 1).

**Table 1:** Primers used in this study

Primer	Sequence (5ʹ to 3ʹ)
FIP	TGGGGTCCATTATCAGACATTTTAGTTTTAGAGTATCATGACGTTCG
BIP	CGAAATGCACCCCGCATTACCCACTGCGTTCTCCATTC
FLP	TGTTCGTTTAGATGAAATC
BLP	TGGTGGACCCTCAGATTCAA
F3	GTTGTTCGTTCTATGAAGACT
B3	GACGTTGTTTTGATCGCG

Features: FIP: Forward inner primer; BIP: backward inner primer; FLP: forward loop primer; BLP: backward loop primer; F3: forward primer; B3: backward primer

### Analytical sensitivity and specificity

To test the analytical sensitivity of the phenol red RT-LAMP assay, a recombinant plasmid carrying the *N* gene was constructed. F3 and B3 primers were used to amplify the *N* gene from a synthetic fragment (Sangon Biotec Co., Ltd., Shanghai, China). PCR conditions were as follows: denaturation at 94 °C for 3 min, 30 cycles at 94 °C for 30 s, at 55 °C for 30 s, and at 72 °C for 30 min, and a final extension step at 72 °C for 10 min. The PCR product was then subjected to 1.5% agarose gel electrophoresis. The amplified gene fragment was purified prior to cloning into the pGEM-T vector (Promega Corporation, Madison, WI) and transformed into TOP10F’ *Escherichia coli* competent cells. Recombinant plasmids were extracted using the Qiagen Spin Miniprep kit (Qiagen, Hilden, Germany) and were sent to Apical Scientific SDN BHD (Kuala Lumpur, Malaysia) for sequencing to confirm their identity. The pGEM-T vector containing the *N* insert was linearized by *BamH*I and transcribed to RNA using RiboMAX™ Large Scale RNA Production Systems (Promega Corporation, Madison, WI) according to the manufacturer’s instructions. The RNA copy number was calculated based on the following formula: copies/µL = 6.02 × 10^23^ × 10^–9^ × concentration (ng/µL)/(fragment length (bp) × 340) [10]. Then, tenfold serial dilutions of the transcribed RNA ranging from 1 × 10^6^ copies/µL to 1 copy/µL were prepared.

Analytical specificity test was performed by using other respiratory viruses such as Adenovirus 4, Coronavirus, Influenza A H3, Influenza B, Novel influenza A H1N1, Parainfluenza 1, Parainfluenza 2, Parainfluenza 3, RSV (subtype A), RSV (subtype B) (AMPLIRUN^®^ Coronavirus RNA control (Vircell Microbiologist, Granada, Spain).

### Clinical sensitivity and specificity test

Clinical sensitivity was evaluated using the formula: (number of true positives)/(number of true positives + number of false negatives), while specificity was calculated as (number of true negatives)/(number of true negatives + number of false positives).

## Results

Analytical sensitivity test of *N* gene for phenol red RT-LAMP was 1 copy/µL RNA. None of the other viruses were detected by the RT-LAMP assay. By using 260 sample here, the clinical sensitivity and specificity of RT-LAMP assay were calculated. We found that most of the RT-PCR positive samples with a Ct < 30 changed colour within the first 20 min of the reaction. Samples with Ct > 30 either took a longer time for the colour to change or there were no changes colour at all. RT-LAMP shows 92.7% sensitivity (95% CI 87.3–96.3%) and 93.6% specificity (95% CI 87.3–97.4%), respectively.

## Discussion

The high cost of currently available commercial RNA extraction kits has impeded mass testing of Covid-19. Therefore, we decided to develop a novel nucleic acid extraction method for SARS-CoV-2 from nasopharyngeal swab samples. Without the use of high throughput equipment, a total of 260 samples were extracted and used as the template for the RT-LAMP assay. We managed to achieve 92.7% sensitivity and 93.6% specificity for RT-LAMP. This extraction method is easy to perform and could be scalable according to the sample size, thereby enabling it to be adopted in both clinical laboratories and field settings. Our newly developed RNA extraction method is much cheaper (USD$2.27/reaction) compared to a commercial QIAamp Viral RNA Mini kit (USD$6.45/reaction). In terms of speed, Chelex extraction method is more rapid (~ 16 min) than the conventional extraction kit (~ 40 min). Also, the commercially available phenol red LAMP mix is costly. For example, WarmStart^®^ Colorimetric LAMP 2× Master Mix (New England Biolabs, United States) costs USD$3.65/reaction. By using the custom make LAMP buffer, one reaction costs only USD$1.95/reaction.

Out of 260 samples tested, results show that RT-LAMP did not detect 11 RT-PCR positive samples. This potentially happened because the viral load of the sample was too low (RT-PCR Ct > 30) and degraded during delivery to our laboratories. These 11 samples were not detected by RT-PCR and RT-LAMP after re-extraction using commercial kit in our laboratories. As mentioned by Azmi et al., SARS-CoV-2 diagnostics is especially challenging during the RNA extraction step, and samples are is often at risk of degradation during delivery [1]. On the other hand, RT-LAMP detected 7 RT-PCR negative samples. These samples were also detected positive by RT-PCR after Chelex extraction. This may be due to insufficient cleaning of the workspace and disinfection of the pipettes. Another possible reason may be cross contamination of samples during the aliquoting stage at the clinical laboratories.

In comparison to our previous study [9], the RT-LAMP assay presented here was using phenol red as the indicator while the previous published RT-LAMP assay employed hydroxynapthol blue (HNB) as the indicator. For positive reaction, additional of HNB into the reaction mix will cause the change of colour from violet to sky blue. Due to the colour changes between positive and negative reaction was not significant, we opted to use phenol red as the indicator. Positive reaction changed from pink to yellow colour while negative reaction remained as pink.

Chelex extraction methods on SARS-CoV-2 have been presented by several groups of researchers previously. However, with minor modifications to the previous presented method by Janíková et al. and Perez et al., the sensitivity of RT-LAMP assay developed here (92.7%) was higher [7, 8]. Flynn et al. reported an RT-LAMP assay with 90% sensitivity by using Chelex extraction protocol [11]. Janíková et al. managed to detect SARS-CoV-2 down to 12 copies/µL while the RT-LAMP assay developed here successfully detected down to 1 copy/µL RNA [7]. Meanwhile, Perez et al. tested the Chelex extracted samples by RT-PCR only, they managed to achieve 84.3% sensitivity of RT-PCR [8].

As for Anathar et al., they reported a direct RT-LAMP assay by using specimens that were either added directly to the reactions, inactivated by a combined chemical and heat treatment step, or inactivated followed by purification with a silica particle-based concentration method. However, we were not able to replicate these methods after several trials. The failure may be due to the different types of VTM being used and presence of inhibitors such as glucose in VTM [12].

We found that increasing incubation time for samples with Ct > 30 was not helpful as nonspecific amplification may occur. This finding was similar as reported by Dao Thi et al. [13]. They found that colorimetric RT-LAMP detection of SARS-CoV-2 is dependent on viral load and showed that positive samples with a RT-PCR Ct < 30 changed colour within the first 30 min of the reaction. Samples with RT-PCR Ct > 30 either took a longer time to change colour (> 35 min) or did not change colour.

We chose a pH dye indicator as the end point detection method as phenol red is cheap and non-toxic for visual detection. Moreover, the colour changes between positive and negative reaction are obvious, and the colour change can be visualized with the naked eye. The distinct colour changes would be useful for people working in the diagnostics field to interpret the COVID-19 results accurately without additional assistance and special equipment. Hence, phenol red has gained popularity among investigators around the world in the development of a diagnostic tool for SARS-CoV-2 [4, 14, 15].

Since phenol red is sensitive to pH changes, the in-house prepared 10× low strength buffer is strongly suggested to be prepared in small aliquots and stored at − 20 °C. It is not recommended to freeze–thaw the buffer too many times to avoid pH changes. Also, RNA was suggested to be eluted in Tris-ethylenediaminetetraacetic acid (TE) buffer pH 8 instead of double distilled water as double distilled water may adsorb carbon dioxide from atmosphere, causing the pH of the water to become slightly acidic. Because of pH buffer changes, false positives may occur.

To enhance the performance of RT-LAMP assay, GuHCl was added. As recommended by Zhang et al., 40 mM of GuHCl was added into the reaction mixture [16]. We noticed that the amplification time was shorten by ~ 5 min compared to samples without GuHCl. No betaine was added in this RT-LAMP assay. Compared to reactions with betaine, we found that the amplification time for reactions without betaine was shortened by ~ 15 min. Similar findings were reported from Fu et al. and García-Bernalt Diego et al. [17, 18]. We believe that our investigation will provide a new pathway to establish a RT-LAMP assay for rapid detection of SARS-CoV-2 and other viruses as well.

For cost/reaction, the RT-LAMP assay combined with Chelex extraction method presented here was way cheaper than commercially RT-LAMP kit and RNA extraction kit. The total cost (Chelex extraction method and custom make LAMP buffer) was USD$4.20/reaction. Meanwhile, the total cost of commercially available LAMP kit such as WarmStart^®^ Colorimetric LAMP 2× Master Mix (New England Biolabs, United States) and QIAamp Viral RNA Mini kit (Qiagen, Hilden. Germany) was USD$10.10/reaction.

## Conclusions

We present a simple RNA extraction procedure from nasopharyngeal swab samples here. By additional of pH indicator dye and additive into the RT-LAMP assay, we managed to develop a rapid, cost effectively and simple-to-interpret assay for the detection of SARS-CoV-2. Therefore, this RT-LAMP assay is speculated to be deployed for mass screening applications in local and referral laboratories.

### Supplementary Information

**Additional file 1.** Result of RT-qPCR and RT-LAMP for 260 samples.

## Data Availability

All relevant data are within the paper or Additional file 1. The information of cloned *N* gene is available at https://www.ncbi.nlm.nih.gov/nuccore/ON911925
