# Supplementary material for: Clinical testing on SARS-CoV-2 swab samples using reverse-transcription loop-mediated isothermal amplification (RT-LAMP)
Source: BMC Infect Dis. 2022 Aug 18;22:697. doi: 10.1186/s12879-022-07684-w (PMC9387413; doi:10.1186/s12879-022-07684-w)
Supplement: Supplementary file 1 — Additional file 1. Result of RT-qPCR and RT-LAMP for 260 samples. [file 12879_2022_7684_MOESM1_ESM.docx]

**Table S1**

| **Sample** | **RT-qPCR** | **RT-LAMP** |
| --- | --- | --- |
| WC480150/21 | 20.69 | 40 |
| WC479481/21 | 21.06 | 30 |
| WC479437/21 | 20.88 | 30 |
| WC479117/21 | 30.32 | 60 |
| WC479549/21 | 34.93 | Negative |
| WC479477/21 | 22.85 | 30 |
| WC479476/21 | 25.52 | 30 |
| WC479461/21 | 15.10 | 30 |
| WC479762/21 | 25.05 | 30 |
| WC479798/21 | 30.19 | 60 |
| WC480571/21 | 28.79 | 40 |
| WC480546/21 | 30.24 | 30 |
| WC480545/21 | 14.33 | 40 |
| WC480544/21 | 14.48 | 40 |
| WC480543/21 | 19.02 | 40 |
| WC480540/21 | 24.72 | 40 |
| WC480535/21 | 16.62 | 30 |
| WC480534/21 | 14.69 | 30 |
| WC480567/21 | Negative | Negative |
| WC480568/21 | Negative | Negative |
| WC480569/21 | Negative | Negative |
| WC480570/21 | Negative | Negative |
| WC480572/21 | Negative | Negative |
| WC480573/21 | Negative | Negative |
| WC480574/21 | Negative | Negative |
| WC480575/21 | Negative | Negative |
| WC480576/21 | Negative | Negative |
| WC480577/21 | Negative | Negative |
| WC480578/21 | Negative | Negative |
| WC480579/21 | Negative | Negative |
| WC481764/21 | 15.17 | 30 |
| WC481905/21 | 20.24 | Negative |
| WC481781/21 | 20.55 | 30 |
| WC481782/21 | 16.81 | 30 |
| WC481785/21 | 15.71 | Negative |
| WC481787/21 | 21.47 | 60 |
| WC481788/21 | 21.38 | 60 |
| WC481868/21 | 29.28 | 60 |
| WC481869/21 | 18.06 | 60 |
| WC481872/21 | 26.11 | 60 |
| WC481873/21 | 28.15 | 60 |
| WC481874/21 | 17.35 | 60 |
| WC481875/21 | 16.11 | 40 |
| WC481876/21 | 17.44 | 30 |
| WC481877/21 | 15.37 | 30 |
| WC481878/21 | 16.49 | 60 |
| WC481879/21 | 18.21 | 60 |
| WC481891/21 | 15.47 | 60 |
| WC481766/21 | Negative | Negative |
| WC481776/21 | Negative | Negative |
| WC481777/21 | Negative | Negative |
| WC481778/21 | Negative | Negative |
| WC481779/21 | Negative | 30 |
| WC481780/21 | Negative | 30 |
| WC481783/21 | Negative | 30 |
| WC481784/21 | Negative | 60 |
| WC481789/21 | Negative | Negative |
| WC481791/21 | Negative | Negative |
| WC481792/21 | Negative | Negative |
| WC481793/21 | Negative | Negative |
| WC485200/21 | 16.16 | 30 |
| WC485201/21 | 19.34 | 30 |
| WC485202/21 | 12.71 | 30 |
| WC485203/21 | 26.25 | 30 |
| WC485205/21 | 25.23 | 30 |
| WC485228/21 | 33.30 | 30 |
| WC485232/21 | 21.52 | 30 |
| WC485236/21 | 17.81 | 40 |
| WC485241/21 | 17.69 | 40 |
| WC485254/21 | 35.69 | 40 |
| WC485274/21 | 13.90 | 40 |
| WC485277/21 | 30.56 | Negative |
| WC485429/21 | 17.18 | 40 |
| WC485480/21 | 31.44 | 60 |
| WC485600/21 | 28.28 | 60 |
| WC485601/21 | 25.08 | 60 |
| WC485602/21 | 17.52 | 60 |
| WC485603/21 | 18.05 | 60 |
| WC485282/21 | Negative | Negative |
| WC485283/21 | Negative | Negative |
| WC485284/21 | Negative | Negative |
| WC485285/21 | Negative | Negative |
| WC485286/21 | Negative | Negative |
| WC485287/21 | Negative | Negative |
| WC485289/21 | Negative | Negative |
| WC485290/21 | Negative | Negative |
| WC485291/21 | Negative | Negative |
| WC485292/21 | Negative | Negative |
| WC485293/21 | Negative | Negative |
| WC485294/21 | Negative | Negative |
| WC492017/21 | 24.22 | 60 |
| WC492018/21 | 23.50 | 60 |
| WC492019/21 | 28.65 | 60 |
| WC492020/21 | 34.56 | 60 |
| WC492021/21 | 31.11 | 30 |
| WC492022/21 | 30.11 | 30 |
| WC492023/21 | 33.65 | 30 |
| WC492024/21 | 23.56 | 60 |
| WC492025/21 | 29.91 | 60 |
| WC492027/21 | 32.20 | 60 |
| WC492028/21 | 33.56 | 60 |
| WC492030/21 | 26.78 | 40 |
| WC492031/21 | 33.87 | 40 |
| WC492032/21 | 35.61 | 40 |
| WC492033/21 | 33.40 | 40 |
| WC492034/21 | 31.22 | 40 |
| WC492035/21 | 26.73 | 40 |
| WC492036/21 | 36.90 | Negative |
| WC492194/21 | Negative | Negative |
| WC492195/21 | Negative | Negative |
| WC492196/21 | Negative | Negative |
| WC492210/21 | Negative | Negative |
| WC492211/21 | Negative | Negative |
| WC492241/21 | Negative | Negative |
| WC492242/21 | Negative | Negative |
| WC492243/21 | Negative | Negative |
| WC492346/21 | Negative | Negative |
| WC492347/21 | Negative | Negative |
| WC492365/21 | Negative | Negative |
| WC492366/21 | Negative | Negative |
| WC499201/21 | 30.11 | 60 |
| WC499205/21 | 31.17 | Negative |
| WC499206/21 | 20.21 | 60 |
| WC499208/21 | 23.22 | 60 |
| WC499230/21 | 26.88 | 40 |
| WC499231/21 | 26.78 | 40 |
| WC499232/21 | 28.79 | 40 |
| WC499233/21 | 29.98 | 40 |
| WC499234/21 | 32.29 | 40 |
| WC499235/21 | 33.19 | 40 |
| WC499236/21 | 24.68 | 40 |
| WC499238/21 | 31.42 | Negative |
| WC499243/21 | 19.20 | 40 |
| WC499245/21 | 23.35 | 40 |
| WC499329/21 | 22.10 | 40 |
| WC499331/21 | 34.32 | 40 |
| WC499346/21 | 31.30 | 40 |
| WC499776/21 | 30.58 | 40 |
| WC499223/21 | Negative | Negative |
| WC499224/21 | Negative | Negative |
| WC499225/21 | Negative | Negative |
| WC499226/21 | Negative | Negative |
| WC499227/21 | Negative | Negative |
| WC499240/21 | Negative | Negative |
| WC499241/21 | Negative | Negative |
| WC499242/21 | Negative | Negative |
| WC499259/21 | Negative | Negative |
| WC499260/21 | Negative | Negative |
| WC499261/21 | Negative | Negative |
| WC499262/21 | Negative | Negative |
| N930 | 25.4 | 60 |
| N944 | 32.71 | 60 |
| N957 | 29.49 | 60 |
| N958 | 34.82 | 40 |
| N967 | 27.35 | Negative |
| N980 | 37.01 | 30 |
| N981 | Negative | Negative |
| N982 | Negative | 60 |
| N983 | 30.27 | 40 |
| N984 | Negative | 40 |
| N985 | Negative | Negative |
| N986 | Negative | Negative |
| N987 | Negative | Negative |
| N992 | 29.51 | 30 |
| N993 | 28.68 | 30 |
| N994 | 34.97 | Negative |
| N995 | Negative | Negative |
| N996 | Negative | Negative |
| N997 | Negative | Negative |
| N998 | Negative | Negative |
| N999 | 26.55 | 60 |
| N1002 | 24.54 | 30 |
| N2102 | 24.61 | 40 |
| N2116 | 24.65 | 30 |
| N2163 | Negative | Negative |
| N2164 | Negative | Negative |
| N2231 | Negative | Negative |
| N2232 | Negative | Negative |
| N2233 | 24.98 | 30 |
| N2234 | Negative | Negative |
| N2235 | Negative | Negative |
| N2236 | Negative | Negative |
| N2237 | Negative | Negative |
| N2238 | Negative | Negative |
| N2239 | 20.18 | 30 |
| N2240 | 19.48 | 30 |
| N2241 | 18.68 | 30 |
| N2242 | 19.93 | 40 |
| N2243 | 22.69 | 20 |
| N2244 | Negative | Negative |
| N2259 | Negative | Negative |
| N2261 | 33.34 | 60 |
| N2262 | 18.00 | 20 |
| N2280 | Negative | Negative |
| N3211 | 34.90 | 60 |
| N3212 | 28.05 | 30 |
| N3315 | 32.11 | 60 |
| N3221 | 23.73 | 60 |
| N3223 | 21.31 | 20 |
| N3226 | 26.46 | 20 |
| N3228 | 31.11 | 20 |
| N3229 | 26.30 | 20 |
| N3338 | 21.71 | 20 |
| N3341 | 25.00 | 20 |
| N3345 | 20.78 | 20 |
| N3349 | 27.69 | 20 |
| N3355 | 34.78 | 30 |
| N3368 | Negative | Negative |
| N3370 | 37.60 | 60 |
| N3384 | Negative | Negative |
| N3398 | Negative | Negative |
| N3399 | Negative | Negative |
| N3401 | Negative | Negative |
| N3400 | Negative | Negative |
| N3405 | Negative | Negative |
| N3407 | Negative | Negative |
| N4192 | 33.20 | 40 |
| N4193 | 30.18 | 20 |
| N4218 | 29.78 | 40 |
| N4219 | 26.45 | 20 |
| N4225 | 28.88 | 40 |
| N4226 | 29.32 | 20 |
| N4227 | 31.21 | 40 |
| N4228 | 32.85 | 40 |
| N4244 | Negative | Negative |
| N4245 | Negative | Negative |
| N4252 | 25.19 | 20 |
| N4253 | Negative | 60 |
| N4254 | 32.88 | 30 |
| N4255 | Negative | Negative |
| N4256 | Negative | Negative |
| N4258 | Negative | Negative |
| N4259 | Negative | Negative |
| N4260 | Negative | Negative |
| N4261 | Negative | Negative |
| N4262 | Negative | Negative |
| N4277 | 30.56 | 20 |
| N4290 | 26.21 | 20 |
| N4386 | 22.62 | 40 |
| N4397 | 22.77 | 40 |
| N4416 | 38.80 | Negative |
| N4419 | 27.53 | 40 |
| N4438 | 22.66 | 20 |
| N4439 | 35.57 | 30 |
| N4440 | 22.80 | 20 |
| N4441 | 21.33 | 20 |
| N4443 | Negative | Negative |
| N4444 | 35.40 | 40 |
| N4448 | 35.12 | 40 |
| N4450 | 37.91 | Negative |
| N4451 | Negative | Negative |
| N4452 | Negative | Negative |
| N4453 | Negative | Negative |
| N4454 | Negative | Negative |
| N4455 | Negative | Negative |
| N4456 | Negative | Negative |
| N4457 | Negative | Negative |
| N4458 | Negative | Negative |
| N4459 | Negative | Negative |
| N4463 | 30.66 | 40 |

Abbrevation: RT-qPCR: real-time reverse transcription polymerase chain reaction;

RT-LAMP: reverse-transcription loop-mediated isothermal amplification
